# Assessment of Oncologists’ Perspectives on Omission of Sentinel Lymph Node Biopsy in Women 70 Years and Older With Early-Stage Hormone Receptor–Positive Breast Cancer

**DOI:** 10.1001/jamanetworkopen.2022.28524

**Published:** 2022-08-24

**Authors:** Christina A. Minami, Ava F. Bryan, Rachel A. Freedman, Anna C. Revette, Mara A. Schonberg, Tari A. King, Elizabeth A. Mittendorf

**Affiliations:** 1 Division of Breast Surgery, Department of Surgery, Brigham and Women’s Hospital, Boston, Massachusetts; 2 Breast Oncology Program, Dana-Farber Brigham Cancer Center, Boston, Massachusetts; 3 Center for Surgery and Public Health, Brigham and Women’s Hospital, Boston, Massachusetts; 4 Department of Surgery, University of Chicago, Chicago, Illinois; 5 Medical Oncology, Dana-Farber Cancer Institute, Boston, Massachusetts; 6 Survey and Qualitative Methods Core, Dana-Farber Cancer Institute, Boston, Massachusetts; 7 General Medicine and Primary Care, Beth Israel Deaconess Medical Center, Boston, Massachusetts

## Abstract

**Importance:**

Randomized clinical trial data have demonstrated that omission of surgical axillary evaluation does not affect overall survival in women 70 years and older with early-stage (clinical tumor category 1 [cT1] with node-negative [N0] disease) hormone receptor (HR)–positive and erb-B2 receptor tyrosine kinase 2 (*ERBB2*; formerly *HER2*)–negative breast cancer. Therefore, the Choosing Wisely initiative has recommended against routine use of sentinel lymph node biopsy (SLNB) in this population; however, retrospective data have revealed that more than 80% of patients eligible for SLNB omission still undergo the procedure. Multidisciplinary factors involved in these patterns remain unclear.

**Objective:**

To describe surgical, medical, and radiation oncologists’ perspectives on omission of SLNB in women 70 years and older with cT1N0 HR-positive, *ERBB2*-negative breast cancer.

**Design, Setting, and Participants:**

This qualitative study used in-depth semi-structured interviews to explore the factors involved in oncologists’ perspectives on providing care to older women who were eligible for SLNB omission. Purposive snowball sampling was used to recruit a sample of surgical, medical, and radiation oncologists representing a wide range of practice types and number of years in practice in the US and Canada. A total of 29 oncologists who finished training and were actively treating patients with breast cancer were interviewed. Interviews were conducted between March 1, 2020, and January 17, 2021.

**Main Outcomes and Measures:**

Recordings from semi-structured interviews were transcribed and deidentified. Thematic analysis was used to identify emergent themes.

**Results:**

Among 29 physicians (16 women [55.2%] and 13 men [44.8%]) who participated in interviews, 16 were surgical oncologists, 6 were medical oncologists, and 7 were radiation oncologists. Data on race and ethnicity were not collected. Participants had a range of experience (median [range] years in practice, 12.0 [0.5-30.0]) and practice types (14 academic [48.3%], 7 community [24.1%], and 8 hybrid [27.6%]). Interviews revealed that the decision to omit SLNB was based on nuanced patient- and disease-level factors. Wide variation was observed in oncologists’ perspectives on SLNB omission recommendations and supporting data. In addition, participants’ statements suggested that the multidisciplinary nature of cancer care may increase oncologists’ anxiety regarding SLNB omission.

**Conclusions and Relevance:**

In this study, findings from interviews revealed that oncologists' perspectives may have implications for the largely unsuccessful deimplementation of SLNB in women 70 years and older with cT1N0 HR-positive, *ERBB2*-negative breast cancer. Interventions aimed at educating physicians, improving patient-physician communication, and facilitating preoperative multidisciplinary conversations may help to successfully decrease SLNB rates in this patient population.

## Key Points

**Question:**

What are surgical, medical, and radiation oncologists’ perspectives on the omission of sentinel lymph node biopsy (SLNB) in women 70 years and older with early-stage hormone receptor–positive breast cancer?

**Findings:**

In this qualitative study involving semi-structured interviews of 29 surgical, medical, and radiation oncologists, the decision to omit SLNB involved nuanced patient- and disease-level factors. Wide variation was observed in oncologists’ perspectives on SLNB omission recommendations and supporting data, and participants’ statements suggest that the multidisciplinary nature of cancer care may increase oncologists’ anxiety regarding omission of SLNB.

**Meaning:**

This study’s findings suggest that interventions aimed at educating physicians, facilitating preoperative multidisciplinary conversations, and improving patient-physician communication may help to appropriately decrease SLNB rates.

## Teaser

This qualitative study describes surgical, medical, and radiation oncologists’ perspectives on the omission of sentinel lymph node biopsy among women 70 years and older with early-stage hormone receptor–positive breast cancer.

## Introduction

More than 30% of new breast cancer diagnoses in the US are among women 70 years and older,^1^ with approximately 80% having hormone receptor (HR)–positive erb-B2 receptor tyrosine kinase 2 (*ERBB2*; formerly *HER2*)–negative disease.^2^ Randomized clinical trial data have demonstrated that omission of surgical axillary staging in women 70 years and older with early-stage (clinical tumor category 1 [cT1] with node-negative [N0] disease) HR-positive, *ERBB2*-negative disease does not result in a survival disadvantage.^3,4,5^ Three clinical trials have specifically explored this area: (1) the Cancer and Leukemia Group B 9343 clinical trial,^3^ which randomized patients 70 years and older with cT1N0 estrogen receptor–positive disease to receive radiotherapy and tamoxifen vs tamoxifen only, with axillary lymph node dissection (ALND) omitted in more than 60% of participants in both arms; (2) the International Breast Cancer Study Group 10-93 clinical trial,^5^ which was originally designed to evaluate disease-free survival equivalence among women 60 years and older with cN0 operable breast cancer who did vs did not receive ALND; and (3) a clinical trial conducted by Martelli et al,^4^ which randomized women aged 65 to 80 years with cT1N0 cancer undergoing quadrantectomy and radiotherapy to receive ALND vs no ALND. None of these clinical trials showed a survival difference between the 2 arms, and axillary recurrence rates among women who did not receive ALND ranged from 3% to 6%.^3,4,5^ In addition, although sentinel lymph node biopsy (SLNB) is viewed as a relatively low-risk procedure, bleeding, infection, increased operative time, seroma, persistent paresthesia, and lymphedema risks persist.^6^ Therefore, the Choosing Wisely initiative recommends against the use of routine SLNB in this patient population,^7^ but use rates remain higher than 80% in Canada and the US.^8,9^

The reasons for the lack of appropriate deescalation of local therapy have not been well studied. Nodal status has traditionally played an important role in adjuvant treatment decisions among medical and radiation oncologists, and a previous study^10^ of SLNB omission in older adults identified multidisciplinary dynamics as a possible factor involved in persistently high SLNB rates. Despite these findings, it remains unclear how the different perspectives of 3 important physician stakeholders in these discussions (surgical, medical, and radiation oncologists) play a role in SLNB rates among this patient population. The objective of this qualitative study was to understand these subspecialists’ perspectives on SLNB omission and to identify specific practice factors that may be involved in deimplementation of SLNB in women 70 years and older with cT1N0 HR-positive, *ERBB2*-negative breast cancer.

## Methods

### Study Design and Setting

To broadly explore oncologists’ attitudes regarding omission of SLNB in patients 70 years and older with cT1N0 HR-positive, *ERBB2*-negative breast cancer, this qualitative study used in-depth semi-structured interviews among a sample of surgical, medical, and radiation oncologists in the US and Canada. Oncologists were eligible for participation if they had finished training and were actively treating patients with breast cancer. The semi-structured interview guide was developed using domains described by the Tailored Implementation for Chronic Diseases checklist,^11^ a screening tool used to identify clinical practice factors; the interview guide was customized for the specialties of interest (Supplement). This study was approved by the institutional review board of the Dana-Farber Cancer Institute. All participants provided verbal informed consent, including consent to use their quotes for publication, before they were interviewed. The study followed the Consolidated Criteria for Reporting Qualitative Research (COREQ) reporting guideline for qualitative studies.^12^

### Interview Participants

The first participants identified were surgical, radiation, and medical oncologists who had practiced in academic, community, or hybrid settings in the Dana-Farber Cancer Institute network within the past 5 years. A snowball strategy was used to recruit additional participants. All participants were recruited via email. Purposive snowball sampling was used to ensure participants were diverse with respect to length of time in practice, practice location, and practice type (Table 1). Interviews continued until thematic saturation (the point at which new themes infrequently emerged and code definitions remained stable) was reached.^13^

**Table 1.** Participant Demographic Characteristics

Characteristic	Participants, No./total No. (%)
Total participants, No.	29
Female	16/29 (55.2)
Male	13/29 (44.8)
**Surgical oncologists**	
Total participants	16/29 (55.2)
Years in practice, median (range)	13.0 (0.5-33.0)
Practice location	
US	
Midwest	4/16 (25.0)
North	2/16 (12.5)
South	4/16 (25.0)
West	4/16 (25.0)
Canada	2/16 (12.5)
Practice type	
Academic	7/16 (43.8)
Community	5/16 (31.3)
Hybrid	4/16 (25.0)
Percentage range of physician’s practice comprising patients with breast cancer diagnoses	10-100
Fellowship training	13/16 (81.3)
Defined clinically node-negative by physical examination alone	8/16 (50.0)
Defined clinically node-negative by physical examination and ultrasonographic findings	8/16 (50.0)
**Medical oncologists**	
Total participants	6/29 (20.7)
Years in practice, median (range)	8.5 (4.0-20.0)
Practice location	
US	
Midwest	0
North	4/6 (66.7)
South	1/6 (16.7)
West	1/6 (16.7)
Canada	0
Practice type	
Academic	4/6 (66.7)
Community	2/6 (33.3)
Hybrid	0
Percentage range of physician’s practice comprising patients with breast cancer diagnoses	20-100
Fellowship training	6/6 (100)
**Radiation oncologists**	
Total participants	7/29 (24.1)
Years in practice, median (range)	18.0 (4.0-36.0)
Practice location	
US	
Midwest	1/7 (14.3)
North	2/7 (28.6)
South	2/7 (28.6)
West	1/7 (14.3)
Canada	1/7 (14.3)
Practice type	
Academic	3/7 (42.9)
Community	0
Hybrid	4/7 (57.1)
Percentage range of physician’s practice comprising patients with breast cancer diagnoses	10-100
Fellowship training	0

### Interview Procedures

Interviews were conducted via telephone between March 1, 2020, and January 17, 2021, by the principal investigator (C.A.M.), a female surgical oncologist with training in qualitative methods. The interviewer had previous professional relationships with 2 participants. All participants knew the interviewer’s professional background. The domains from the Tailored Implementation for Chronic Diseases checklist^11^ included in this study were guideline factors; social, political, and legal factors; professional interactions; individual health professional factors; and patient factors. Because this study was designed to focus on different multidisciplinary perspectives, separate interview guides were developed for surgical oncologists (eAppendix 1 in the Supplement) and medical or radiation oncologists (eAppendix 2 in the Supplement). The 2 interview guides were developed over several iterations, with input from a qualitative research expert (A.C.R.) and oncologists (R.A.F., T.A.K., and E.A.M.), to ensure content and face validity and to evaluate the approximate time for interview completion. The interview guides were also pilot tested among 3 physicians (1 surgical oncologist, 1 medical oncologist, and 1 radiation oncologist), with minor resultant modifications. No changes to the guides were made during the subsequent interviews. Interviews were digitally recorded, with a mean duration of 15.7 minutes (range, 7.0-47.0 minutes). Demographic information regarding participant sex and practice characteristics were obtained at the beginning of each interview. Notes taken during each interview were also documented by the interviewer. Incentives in the form of $50 gift cards were provided, although they were not accepted by all participants.

### Statistical Analysis

Digital audio recordings were transcribed verbatim and deidentified. After all data were collected and reviewed, thematic analysis was conducted using an iterative, multistage process that incorporated both inductive and deductive coding. We applied deductive codes based on the domains of the Tailored Implementation for Chronic Diseases checklist,^11^ then expanded the codebook to include inductive codes that emerged from the data. The initial codebook was created and refined by 3 team members (C.A.M., A.F.B., and A.C.R.). Each transcript was then coded by 2 team members (C.A.M. and A.F.B.) using NVivo software, version 12.5.0 (QSR International); these team members (C.A.M. and A.F.B.) met frequently to resolve areas of disagreement and reach consensus in coding. After all data were coded, the research team (C.A.M., A.F.B., and A.C.R.) met to discuss the salience of emergent themes. Codes were analyzed both within and across subgroups (ie, surgical, medical, and radiation oncologists).

## Results

Over the study period, 51 surgical, medical, and radiation oncologists were identified as potential participants by colleagues; of those, 29 oncologists consented to participate. Of the 22 physicians who did not respond to the invitation to participate, 12 were surgical oncologists, 6 were medical oncologists, and 4 were radiation oncologists.

Of the 29 oncologists who chose to participate, 16 (55.2%) were surgeons, 6 (20.7%) were medical oncologists, and 7 (24.1%) were radiation oncologists (Table 1). A total of 16 participants (55.2%) were women, and 13 participants (44.8%) were men. Data on race and ethnicity were not collected. Most surgical oncologists (13 physicians [81.3%]) and medical oncologists (6 physicians [100%]) had fellowship training, whereas none of the radiation oncologists had fellowship training, which reflects the nature of their specialty. Overall, participants had a range of experience (median [range] years in practice, 12.0 [0.5-30.0]) and practice types (14 academic [48.3%], 7 community [24.1%], and 8 hybrid [27.6%]). Years of experience ranged from 6 months to 36 years (median [range] for surgical oncologists: 13.0 [0.5-33.0] years; medical oncologists: 8.5 [4.0-20.0] years; radiation oncologists: 18.0 [4.0-36.0] years). All 3 specialties were represented in each region, with the exception of medical oncologists in the Midwest and Canada. Each specialty had academic practice representation (7 surgical oncologists [43.8%], 4 medical oncologists [66.7%], and 3 radiation oncologists [42.9%]), and 2 specialties had community practice representation (5 surgical oncologists [31.3%] and 2 medical oncologists [33.3%]). The estimated percentage of physicians’ practices comprising patients with breast cancer diagnoses ranged from 10% to 100% among surgical and radiation oncologists and from 20% to 100% among medical oncologists.

### Factors Involved in the Decision to Omit SLNB

Participants identified a variety of factors integral to the decision to omit SLNB in this patient population. Tumor characteristics associated with worse prognosis (eg, larger tumor size, presence of lymphovascular invasion, low estrogen receptor positivity, or higher grade) and higher likelihood of a positive node discouraged SLNB omission; better prognostic factors and lower likelihood of a positive node encouraged SLNB omission (Table 2). The likelihood that a patient would receive adjuvant systemic therapies (eg, endocrine therapy adherence or chemotherapy receipt) was also important to oncologists’ comfort level with SLNB omission.

**Table 2.** Representative Quotes About Factors Involved in the Decision to Omit SLNB

Theme	TICD checklist domain	Representative quote
Tumor factors are important	Individual health professional factors	“I go by the pathology features. For example, for patients with extensive LVI…a relative low ER and negative PR” (medical oncologist).
Oncologists’ opinions on the likelihood of patient receipt of adjuvant systemic therapies are important	Individual health professional factors	“The key question is whether they’ll have chemotherapy or not, whether they can tolerate it” (surgical oncologist).
		“…And then we think, is she going to be taking endocrine therapy....That’ll give us just that little bit more reassurance that she doesn’t need more local therapy” (medical oncologist).
Information from other modalities can make oncologists more comfortable with SLNB omission	Individual health professional factors	“I think with the oncotype in the elderly women 70 or above, a lot of times we do omit the lymph node dissection…[if the patient has] a preoperative oncotype and it’s favorable, it’s not gonna influence whether they’re gonna be a candidate for systemic therapy” (surgical oncologist).
		“Most if not all women get an axillary ultrasound as part of their presurgical workup. Most of them also get an MRI” (radiation oncologist).
SLNB omission can have consequences for treatment decision-making	Individual health professional factors	“[Without a sentinel lymph node biopsy] I might do high tangents, and if there were really bad features of the tumor, I might even consider comprehensive nodal irradiation” (radiation oncologist).
		“Some people may feel somewhat uncomfortable offering partial breast irradiation to someone who hasn’t had an axillary nodal assessment” (radiation oncologist).
Physiological age is important	Patient factors	“I think [we have to treat] the patient from a broader perspective—from a physiologic standpoint as opposed to just saying everyone over 70 gets treated the same way” (surgical oncologist).
		“My understanding is that data was often based on age alone as the primary variable. And my concern with a blanket statement on age alone is that chronological age is not the same as physical and functional” (medical oncologist).
Chronological age cutoffs are important	Patient factors	“All the surgical oncologists will…omit sentinel lymph node biopsies for women older than 75 years old” (medical oncologist).
		“The real big cutoff is—the biggest cutoff is 85 and older…that’s when things really catch up to you” (radiation oncologist).
Formal geriatric assessment is not routinely performed	Patient factors	“But a lot of it really does for me have to do with eyeballing patients. I have patients who are in a nursing home setting and they come to me in a wheelchair or something and they’re brought by someone. And it’s very easy to look at these patients and say, you know, we really don’t need sentinel nodes in these patients” (surgical oncologist).
		“A long time ago we tried [geriatric assessments], but it was just too time consuming…I personally tried administering it like a couple of times, and I’m like, no, this is not practical” (medical oncologist).
Patient preference may play a primary role in decisions	Patient factors	“I would say about 80% of the patients will opt to do it…because they want to be treated like they are 40 years old and second is because the radiation oncologist, they would rather avoid radiation” (surgical oncologist).
		“A lot of it is driven by—not that [patients have] necessarily done their own research, but I think they’re influenced by maybe other friends, family members” (radiation oncologist).
Oncologists’ approach to the SLNB omission conversation varies	Patient factors	“When I talk to these patients about avoiding sentinel node biopsy, I always preface by saying that standard of care is sentinel node biopsy” (surgical oncologist).
		“And what I think we’re not doing a good job is explaining why this might not be standard of care but might be the right individualized, personalized approach for you. So, it comes down to how we communicate our decisions with them when we are making them” (medical oncologist).
SLNB omission does not have consequences for patient outcomes	Patient factors	“Zero [consequences for cancer outcomes]…and in fact, I would say if you can give a negative number, I would give a negative number, really” (surgical oncologist).
		“The thing that I worry most about is, I’ve seen a few patients who did not have full treatment, and even with treatment, I’ve seen it, too. A high axillary or superclav failure that’s entwined with the vessels and a great deal of plexus, and it’s deemed inoperable. And then you’re—you’ve got this person in front of you now with an incurable, basically, cancer” (radiation oncologist).
Financial considerations may have implications for decision-making	Social, political, and legal factors	“Definitely no financial incentives, no malpractice concerns as well” (surgical oncologist).
		“There certainly is a financial implication because you do get reimbursed for a sentinel lymph node biopsy when you’re RVU based” (surgical oncologist).
Malpractice considerations may have implications for decision-making	Social, political, and legal factors	“I think malpractice is the reason why it is done in the first place…you don’t want to be the doc who missed the axilla” (medical oncologist).

Abbreviations: ER, estrogen receptor; LVI, lymphovascular invasion; MRI, magnetic resonance imaging; PR, progesterone receptor; RVU, relative value unit; SLNB, sentinel lymph node biopsy; TICD, Tailored Implementation for Chronic Diseases.

Important patient-level factors included geriatric-specific concerns, such as comorbidities and physiological age. Across specialties, older physiological age and presence of comorbidities encouraged SLNB omission; however, despite the importance oncologists placed on consideration of geriatric-specific concerns, validated geriatric screening tools were used by only 1 participant. Participants stated that they relied on “eyeball” assessment. Perspectives on chronological age were a major subtheme, with many participants stating that 70 years was too young a cutoff to automatically omit SLNB. There was a lack of consensus regarding a more reasonable threshold, with ages 75 years, 80 years, and 85 years mentioned as potential alternatives.

Patient preference was also perceived as an important consideration, but there was a wide range of understanding of patient views regarding SLNB omission. Although some participants thought that “less is more” with regard to SLNB use among older patients, others believed that most older women would want to receive SLNB either because they wanted to be treated like younger patients or they wanted peace of mind. Oncologists perceived patient educational level, views of family and friends, and patient anxiety level as being potentially influential for patients’ views.

Although all participants agreed that SLNB omission did not have consequences for survival, several radiation oncologists expressed anxiety regarding regional recurrences. In addition, radiation oncologists commented that, although SLNB omission may not necessarily change their decision regarding whether to provide treatment with radiotherapy, omission might change the type of radiotherapy (eg, addition of high tangents, addition of comprehensive nodal radiotherapy, or whole breast vs partial breast radiotherapy) they would provide. Surgical oncologists were concerned about the potential consequences of SLNB omission for systemic therapy decisions; however, medical oncologists said they did not experience undue difficulties in making chemotherapy recommendations without SLNB results. Factors that were disease specific and external to the patient, such as legal or financial issues, were mentioned; however, participants disagreed about whether these factors had any substantial implications for decision-making.

### Perspectives on SLNB Omission Recommendations and Supporting Data

Participants across disciplines stated that the Choosing Wisely recommendation led them to consider SLNB omission; however, perspectives varied regarding the lack of specificity of the recommendation (which states, “do not routinely use SLNB in clinically node-negative women ≥70 years of age with early-stage HR+, *HER2-* invasive breast cancer”^7^) (Table 3). Although some participants approved of the wording because it reflected the complexities inherent in medical decision-making, 1 surgical oncologist said that the “wishy-washy” nature of the wording left too much open to interpretation. Institutional guidelines that addressed this lack of specificity (eg, integration of tumor size and grade into rules for SLNB omission or practice of unconditional SLNB omission among those aged ≥75 years) were identified by a subset of participants.

**Table 3.** Representative Quotes About Perspectives on SLNB Omission Recommendation and Supporting Data

Theme	TICD checklist domain	Representative quote
Perspectives vary on the lack of specificity of the Choosing Wisely recommendation	Guideline factors	“It’s a little wishy-washy…it leaves a lot of wiggle room now, especially for the nonsurgeons to say, well, it’s not saying don’t do it” (surgical oncologist).
		“I think the particular recommendation…it’s quite nuanced, which I really like. It’s not definitive” (surgical oncologist).
		“I’m totally good with guidelines as long as everyone takes them with a grain of salt and understand[s] that a human is much more complex than the categories we place them in” (medical oncologist).
Institutional guidelines are more specific than the Choosing Wisely recommendation	Guideline factors	“Institutionally, we have a disease group that agrees on different pathways.…If you’re over 70, T1A through T1C grade 1, we’ll avoid sentinel node biopsy. If you’re a grade 2, it has to be less than 1 centimeter” (surgical oncologist).
		“All the surgical oncologists will…omit sentinel lymph node biopsies for women older than 75 years old. However, between 70 to 74 years old, we don’t want them to make a cookie cutter rather than individual case basis” (medical oncologist).
Supporting data are weak	Guideline factors	“The study that they used is a retrospective SEER database, I think, so we don’t have any prospective studies” (surgical oncologist).
		“[The data are] not terribly terrific…sometimes people will vote with their feet before they really have the data” (radiation oncologist).

Abbreviations: SEER, Surveillance, Epidemiology, and End Results; SLNB, sentinel lymph node biopsy; TICD, Tailored Implementation for Chronic Diseases.

Data supporting SLNB omission were perceived as weak, even among those who favored omission. Although several surgical oncologists were able to cite the randomized clinical trial data supporting the Choosing Wisely recommendation, most participants thought the recommendation was supported by retrospective data. Those who were unfamiliar with the clinical trial data but supported SLNB omission cited an overall understanding that omission was reasonable based on what was already known about the lack of survival benefit of axillary surgical procedures among those with node-negative breast cancer. As 1 surgeon said, “It’s sort of like lap choles…you don’t need a randomized clinical trial to let you know that…the patients recover much more readily than those who have an open cholecystectomy.”.

### Participants’ Comfort Level With SLNB Omission

Themes pertaining to professional interactions highlighted the idea that multidisciplinary care had the potential to complicate physicians’ comfort level with SLNB omission (Table 4). Participants noted the potential for patients to receive mixed messages about the utility of SLNB and its implications for treatment choices. Notably, the potential for miscommunication provoked physician anxiety, with 1 radiation oncologist stating, “the patients get really, really upset when there seems to be a lack of consensus among the providers.”

**Table 4.** Representative Quotes About Physicians’ Comfort Level With SLNB Omission

Theme	TICD checklist domain	Representative quote
Potential miscommunication provokes physician anxiety	Professional interactions	“…If you don’t work closely with your radiation and medical oncologists, I feel like there are some radiation doctors who will, if you haven’t adequately staged the axilla, then they might irradiate the axillary lymph node or the nodal basin.…And then, also, I have some medical oncologists that still just really like to have that information, and they’ll mention to the patient that they should have had a sentinel node. So, there will just be, like, some mixed communication, and then the patient feels less confident in the care they’ve received” (surgical oncologist).
SLNB omission can lead to the use of alternative approaches among medical and radiation oncologists	Professional interactions	“[In the absence of the sentinel node], if we were considering chemotherapy, we would send the oncotype” (medical oncologist).
		“[In the absence of an SLNB], I’d probably get an ultrasound. I might even get a CT” (radiation oncologist).
Medical and radiation oncologists defer to surgeons regarding final decision-making	Professional interactions	“I feel like usually the surgeon has a good reason for not wanting to do it in that instance, so I rarely recommend going back and doing a sentinel lymph node” (radiation oncologist).
Multidisciplinary discussions are necessary	Professional interactions	“We certainly review all of our cases through our multidisciplinary conference that we have on a weekly basis. And so, it’s more or less a decision that myself, as the only breast surgeon in the community, and the oncologist sit down and talk about” (surgical oncologist).
		“And sometimes you just have to take a stand and say, eh, well, I’m not going to treat this patient without surgery...frankly, if it’s a team approach, somebody makes a decision without my input initially, that is something that I don’t have to accept blindly” (radiation oncologist).

Abbreviations: CT, computed tomography; SLNB, sentinel lymph node biopsy; TICD, Tailored Implementation for Chronic Diseases.

Although many radiation and medical oncologists expressed deference to surgical oncologists with regard to decision-making, they identified various alternative approaches that could be used when nodal staging information was unavailable. Oncotype diagnosis, additional imaging (ultrasonography, magnetic resonance imaging, and computed tomography), and the Memorial Sloan Kettering sentinel lymph node metastasis nomogram^14^ were all cited as sources of data to aid adjuvant decision-making.

The lack of philosophical consensus on SLNB omission both within and among specialties was highlighted. One surgical oncologist stated, “Some of my medical oncologists…have a certain preference point for me to go a certain way,” whereas another said, “Most medical oncologists and radiation oncologists seem completely accepting and on board with the recommendation. I think where we have the most variation actually in our practice is amongst surgeons.” However, preoperative interdisciplinary discussion, both through formal multidisciplinary tumor boards and less formal physician-to-physician communication, was deemed integral to the decision to omit SLNB.

## Discussion

This qualitative study found that the decision to omit SLNB was based on nuanced patient- and disease-level factors, there was wide variation in oncologists’ perspectives on SLNB omission recommendations and supporting data, and the multidisciplinary nature of cancer care may increase oncologists’ anxiety regarding SLNB omission. Among those findings, several themes suggested there were possible modifiable barriers.

Although oncologists identified patient- and disease-level factors that complicated the decision to omit SLNB in women 70 years and older with HR-positive, *ERBB2*-negative breast cancer, the themes regarding the importance of patient preference and physiological age could be addressed with interventions to improve patient-physician communication. As some participants described, it is possible that patient preference for SLNB is strong, similar to the sentiment that underlies persistently high rates of contralateral prophylactic mastectomy.^15^ It is possible that 1 reason for the successful deescalation of axillary surgical procedures (in accordance with the findings of the American College of Surgeons Oncology Group Z0011 clinical trial,^16^ in which patients who underwent breast conservation procedures after receipt of only 1 or 2 positive SLNB results did not benefit from completion ALND) is that completion ALND is not routinely offered as a shared decision, whereas SLNB in older adults with early-stage HR-positive cancer may be.^17^ It is also possible that, even though oncologists described engaging in the shared decision-making process, patients may not have perceived that they had a choice. Several studies^17,18^ have reported that older patients with breast cancer did not remember being presented with a choice in treatment and instead only remembered that a recommendation was made. Additional conversational nuance occurs when addressing patient age in this clinical context. Many participants acknowledged the importance of physiological age yet continued to use chronological age in decision-making. Integration of objective geriatric assessments into clinical practice may aid with this problem. However, as 1 surgical oncologist noted, “I find myself awkwardly talking around the life expectancy issue,” suggesting that training physicians in the skillful incorporation of shared decision-making and conversations about physiological age may also be needed to aid deimplementation efforts.

The variation noted in oncologists’ perspectives on SLNB omission recommendations and supporting data also revealed targets for intervention. Although 1 theme was “supporting data are perceived as being weak,” the relatively low level of oncologists’ knowledge of the clinical trial data supporting the Choosing Wisely recommendation, even among participants who supported SLNB omission, suggests this is an area to be addressed. Although some participants thought the recommendation was supported only by retrospective data, randomized clinical trial data support the omission of axillary surgical procedures in older patients with low-risk disease. Participants who supported SLNB omission without knowing the clinical trial data mentioned what was historically known about the utility of axillary evaluation, ostensibly referring to the general principle proposed in large clinical trials such as the National Surgical Adjuvant Breast and Bowel Project B-04 study,^19^ which demonstrated that axillary evaluation among patients with clinically node-negative cancer did not affect survival. However, this lack of specific knowledge pertaining to the practice of SLNB omission in women 70 years and older with cT1N0 HR-positive disease may have implications for treatment conversations between physician and patient. Although we only captured physicians’ perspectives regarding the ways in which this issue is discussed, it is possible that the lack of awareness of data supporting SLNB omission hinders physicians’ ability to facilitate discussion regarding this nuanced issue. Physician distrust of data supporting SLNB omission was also captured in a qualitative study by Smith et al,^10^ which assessed multiple Choosing Wisely recommendations; however, the current study found that the disbelief in data may actually be based on a lack of reliable knowledge. If included as part of a multifaceted approach to deimplementation, educational efforts focused on disseminating the existing clinical trial data to physicians, with endorsement by medical and radiation oncologists, may address this barrier. However, the proportion of physicians who would deem the existing data sufficient to completely accept SLNB omission remains unclear.

The third important finding highlighted the difficulties of navigating multidisciplinary cancer care. Surgical oncologists acknowledged that they did not know whether their surgical decision would ultimately have consequences for adjuvant decision-making and whether medical oncologists would communicate to patients that they should have undergone SLNB. Despite this uncertainty, the medical oncologists in our study did not express undue concern about making systemic treatment recommendations in the absence of nodal staging, suggesting that some surgeons’ anxieties may have been unfounded. However, radiation oncologists stated that a lack of surgical nodal staging might increase regional recurrence risk and could have implications for their treatment planning (eg, keep them from offering partial breast radiotherapy). Participants who did not express concerns about the consequences of SLNB omission for adjuvant treatment planning referred to the importance of preoperative conversations. Although increasing preoperative communication can be readily addressed when practitioners can easily access one another, it is difficult in cases in which patients travel for their surgical care but return to their local communities to receive systemic treatment and radiotherapy consultations. Finding successful and efficient ways to facilitate preoperative treatment conversations could be an important part of a greater deimplementation effort.

### Limitations

This study has several limitations. First, the study was only designed to capture the physician perspective; without the patient perspective, the findings may misrepresent certain aspects of the physician-patient interaction. This issue will be addressed in future work. Second, respondent bias may have been inherent because oncologists who were unaware of the data supporting SLNB omission or noncommittal about the omission of SLNB in the patient population of interest may have been less likely to participate in an interview, thereby skewing our results. Although we were careful to use purposive sampling to ensure a range of years in practice and practice types, we were unable to explore whether the racial and ethnic backgrounds of the physician or patient mix were involved in physician perspectives. Third, although we reached saturation of theme, surgical oncologists were overrepresented in this sample, possibly giving a greater voice to surgeons. However, analysis within and across subspecialties highlighted differences and similarities when present.

## Conclusions

This qualitative study found that oncologists viewed omission of SLNB in women 70 years and older with cT1N0 HR-positive, *ERBB2*-negative breast cancer as a nuanced decision that was based on multiple disease- and patient-level factors and that the multidisciplinary nature of current cancer care may be a complicating factor in surgical decision-making. Interventions aimed at educating physicians, improving patient-physician communication, and facilitating preoperative multidisciplinary conversations may help to decrease SLNB rates in a meaningful and appropriate way.

## Notes

Supplement. **eAppendix 1.** Surgical Oncologist Interview Guide **eAppendix 2.** Medical and Radiation Oncologist Interview Guide

## References

[1] Surveillance, Epidemiology, and End Results Program. Cancer stat facts: female breast cancer. National Cancer Institute; 2022. Accessed July 3, 2022. https://seer.cancer.gov/statfacts/html/breast.html

[2] Howlader N, Altekruse SF, Li CI, . US incidence of breast cancer subtypes defined by joint hormone receptor and HER2 status. J Natl Cancer Inst. 2014;106(5):dju055. doi:10.1093/jnci/dju055 24777111 PMC4580552

[3] Hughes KS, Schnaper LA, Bellon JR, . Lumpectomy plus tamoxifen with or without irradiation in women age 70 years or older with early breast cancer: long-term follow-up of CALGB 9343. J Clin Oncol. 2013;31(19):2382-2387. doi:10.1200/JCO.2012.45.2615 23690420 PMC3691356

[4] Martelli G, Boracchi P, Ardoino I, . Axillary dissection versus no axillary dissection in older patients with T1N0 breast cancer: 15-year results of a randomized controlled trial. Ann Surg. 2012;256(6):920-924. doi:10.1097/SLA.0b013e31827660a8 23154393

[5] Rudenstam CM, Zahrieh D, Forbes JF, ; International Breast Cancer Study Group. Randomized trial comparing axillary clearance versus no axillary clearance in older patients with breast cancer: first results of International Breast Cancer Study Group Trial 10-93. J Clin Oncol. 2006;24(3):337-344. doi:10.1200/JCO.2005.01.5784 16344321

[6] Lucci A, McCall LM, Beitsch PD, ; American College of Surgeons Oncology Group. Surgical complications associated with sentinel lymph node dissection (SLND) plus axillary lymph node dissection compared with SLND alone in the American College of Surgeons Oncology Group Trial Z0011. J Clin Oncol. 2007;25(24):3657-3663. doi:10.1200/JCO.2006.07.4062 17485711

[7] Society of Surgical Oncology. Don’t routinely use sentinel node biopsy in clinically node negative women ≥70 years of age with early stage hormone receptor positive, HER2 negative invasive breast cancer. Choosing Wisely, ABIM Foundation. July 12, 2016. Updated July 27, 2021. Accessed July 3, 2022. https://www.choosingwisely.org/clinician-lists/sso-sentinel-node-biopsy-in-node-negative-women-70-and-over

[8] Louie RJ, Gaber CE, Strassle PD, Gallagher KK, Downs-Canner SM, Ollila DW. Trends in surgical axillary management in early stage breast cancer in elderly women: continued over-treatment. Ann Surg Oncol. 2020;27(9):3426-3433. doi:10.1245/s10434-020-08388-8 32215758 PMC7415703

[9] Thompson J, Le J, Hop A, Melnik M, Paulson J, Wright GP. Impact of Choosing Wisely recommendations on sentinel lymph node biopsy and postoperative radiation rates in women over age 70 years with hormone-positive breast cancer. Ann Surg Oncol. 2021;28(10):5716-5722. doi:10.1245/s10434-021-10460-w 34333704

[10] Smith ME, Vitous CA, Hughes TM, Shubeck SP, Jagsi R, Dossett LA. Barriers and facilitators to de-implementation of the Choosing Wisely guidelines for low-value breast cancer surgery. Ann Surg Oncol. 2020;27(8):2653-2663. doi:10.1245/s10434-020-08285-0 32124126 PMC7338259

[11] Flottorp SA, Oxman AD, Krause J, . A checklist for identifying determinants of practice: a systematic review and synthesis of frameworks and taxonomies of factors that prevent or enable improvements in healthcare professional practice. Implement Sci. 2013;8:35. doi:10.1186/1748-5908-8-35 23522377 PMC3617095

[12] Tong A, Sainsbury P, Craig J. Consolidated criteria for reporting qualitative research (COREQ): a 32-item checklist for interviews and focus groups. Int J Qual Health Care. 2007;19(6):349-357. doi:10.1093/intqhc/mzm042 17872937

[13] Bradley EH, Curry LA, Devers KJ. Qualitative data analysis for health services research: developing taxonomy, themes, and theory. Health Serv Res. 2007;42(4):1758-1772. doi:10.1111/j.1475-6773.2006.00684.x 17286625 PMC1955280

[14] Memorial Sloan Kettering Cancer Center. Breast cancer nomogram: sentinel lymph node metastasis. Memorial Sloan Kettering Cancer Center; 2014. Accessed July 3, 2022. https://nomograms.mskcc.org/Breast/BreastSLNodeMetastasisPage.aspx

[15] Jagsi R, Hawley ST, Griffith KA, . Contralateral prophylactic mastectomy decisions in a population-based sample of patients with early-stage breast cancer. JAMA Surg. 2017;152(3):274-282. doi:10.1001/jamasurg.2016.4749 28002555 PMC5531287

[16] Giuliano AE, Ballman KV, McCall L, . Effect of axillary dissection vs no axillary dissection on 10-year overall survival among women with invasive breast cancer and sentinel node metastasis: the ACOSOG Z0011 (Alliance) randomized clinical trial. JAMA. 2017;318(10):918-926. doi:10.1001/jama.2017.11470 28898379 PMC5672806

[17] Wang T, Mott N, Miller J, . Patient perspectives on treatment options for older women with hormone receptor–positive breast cancer: a qualitative study. JAMA Netw Open. 2020;3(9):e2017129. doi:10.1001/jamanetworkopen.2020.17129 32960279 PMC7509630

[18] Schonberg MA, Birdwell RL, Bychkovsky BL, . Older women’s experience with breast cancer treatment decisions. Breast Cancer Res Treat. 2014;145(1):211-223. doi:10.1007/s10549-014-2921-y 24682710 PMC8370713

[19] Fisher B, Jeong JH, Anderson S, Bryant J, Fisher ER, Wolmark N. Twenty-five–year follow-up of a randomized trial comparing radical mastectomy, total mastectomy, and total mastectomy followed by irradiation. N Engl J Med. 2002;347(8):567-575. doi:10.1056/NEJMoa020128 12192016

