# Supplementary material for: Assessment of Oncologists’ Perspectives on Omission of Sentinel Lymph Node Biopsy in Women 70 Years and Older With Early-Stage Hormone Receptor–Positive Breast Cancer
Source: JAMA Netw Open. 2022 Aug 24;5(8):e2228524. doi: 10.1001/jamanetworkopen.2022.28524 (PMC9403774; doi:10.1001/jamanetworkopen.2022.28524)
Supplement: Supplement. — eAppendix 1. Surgical Oncologist Interview Guide eAppendix 2. Medical and Radiation Oncologist Interview Guide [file jamanetwopen-e2228524-s001.pdf]

Supplementary Online Content

Minami CA, Bryan AF, Freedman RA, et al. Assessment of oncologists' perspectives on omission of sentinel lymph node biopsy in women 70 years and older with early-stage hormone receptor–positive breast cancer. *JAMA Netw Open*. 2022;5(8):e2228524. doi:10.1001/jamanetworkopen.2022.28524

eAppendix 1. Surgical Oncologist Interview Guide

eAppendix 2. Medical and Radiation Oncologist Interview Guide

This supplementary material has been provided by the authors to give readers additional information about their work.

© 2022 Minami CA et al. *JAMA Network Open*.

eAppendix 1. Surgical Oncologist Interview Guide

Introduction

Thank you for taking the time to speak with me today. We are speaking with physicians to better understand their perspectives on axillary evaluations in older women with breast cancer. Our discussion today will help inform a survey that will explore issues and attitudes towards the possible omission of axillary evaluation in older women with early-stage hormone-positive breast cancer. There are no right or wrong answers in our discussion today, as we are here to better understand your experience and perspectives on the topic at hand.

This interview will be recorded and takes, on average, twenty minutes. Recordings will not be shared with your colleagues, patients or families, or with anyone else outside the research team. I want to be sure that you understand that being part of this interview is voluntary, you can opt to not answer questions or end the conversation at any time.

We appreciate your participation in this study.

Obtain informed consent

Do you have any questions before we begin the interview?

Begin recording

I. Demographic Information

First off, could I get some baseline information about you?

1) How many years have you been in practice?

2) How would you describe your practice with regards to being academic or community-based or a hybrid model?

3) In what field/specialty, if any, are you board certified?

4) What fellowship training do you have, if any?

5) What is the scope of your surgical practice?

a) What percentage of your practice is breast surgery?

b) Approximately how many women do you see a month who are over 70, with clinically node-negative, hormone-positive breast cancer?

c) How do you usually clinically evaluate the axilla in these women (i.e. physical exam and/or U/S?)

If surgeon routinely uses U/S, ask how they define being "clinically node-negative"

d) How do you usually surgically evaluate the axilla in these women (i.e. ALND or SLNB)?

If surgeon usually performs ALND, ask for further explanation

For the next set of questions, I am asking you to focus specifically on the management of patients 70 and older, with clinically node-negative, hormone-positive breast cancer.

II. Current Practice

A. Individual Health Professional Factors: Professional Behavior

6) How do you usually approach the decision to evaluate the axillary in this patient population?

© 2022 Minami CA et al. *JAMA Network Open*.

-What clinical and disease factors play a role in your decision to evaluate the axillary?

-With this patient population, how often, if ever, do you consider the omission of the axillary evaluation? In what percentage of patients do you actually omit it?

-How has your approach changed over time?

B. Individual Health Professional Factors: Knowledge/Cognitions

If surgeon does omit axillary evaluation:

7) What do you see as the pros and the cons of omitting axillary evaluations?

-For example, what potential consequences might keep you from omitting axillary evaluation?

8) What are your discussions with patients like around axillary evaluation/omission?

-For example, to what extent do you counsel patients about whether or not to pursue axillary evaluation?

9) Can you describe any situations in which your patient has preferred to proceed with the axillary evaluation even though you offer to omit it?

-How often does this happen?

If surgeon never omits axillary evaluation:

10) Overall, how do you feel about the potential omission of axillary evaluation in this population?

11) Have you come across any data/studies that explore omission of axillary evaluation in this population? If so, what do you think of these data?

C. Patient Factors:

12) From your perspective, what do patients think about omitting SLNB? Is it an overall positive or negative for the patient?

13) What impact do you think this practice has on patient outcomes? [probe on reasons behind the impact]

Possible probe:

-Do you turn to any particular published data that guide your opinions?

D. Social, Political, and Legal Factors

14) Are there any external factors (like financial pressure or incentive, or regulations) that influence your approach to axillary evaluation?

Possible probes:

-Do financial incentives or disincentives play a role?
-Or institutional pressures or regulations?
-What about malpractice environment?

© 2022 Minami CA et al. *JAMA Network Open*.

III. In Theory

A. Individual Health Professional Factors: Cognitions

If surgeon unaware of trial data supporting omission of axillary evaluation:

We are curious about use of axillary evaluation in this patient population because there are data from randomized controlled trials demonstrating that axillary evaluation could be safely omitted in this population without compromising survival, and with an increase in axillary recurrence rate of only 3%. In 2016, the Society of Surgical Oncology, the SSO, released a guideline counseling against routine use of axillary evaluation in this patient population.

15) What are your initial reactions to hearing this information?

-How, if at all, does this information impact your consideration on axillary evaluation?

16) Considering the SSO guidelines, to what extent would you be willing to consider omission of axillary evaluation? [probe on specific reasons]

17) Can you describe any factors or reasons that might keep you from omitting axillary evaluation?

If surgeon aware of trial data supporting omission of axillary evaluation:

The Society of Surgical Oncology's Choosing Wisely 2016 made the following recommendation: "Don't routinely use sentinel node biopsy in clinically node negative women $\geq$70 years of age with hormone positive invasive breast cancer."

18) Can you tell me what you understand the intent of the Choosing Wisely campaign to be?

19) What were your initial reactions to this recommendation upon learning about it?

Possible probes:

-What do you think of the quality of evidence supporting this practice?
[probe on concerns, if any]

-What do you think of the SSO as a source for practice recommendations?
[probe on other sources of practice recommendations that they use]

- What do you think of the clarity of the recommendation?

B. Professional Interactions: Communication and Influence

20) How would you describe the communication you have, if any, regarding axillary management in this population of patients with your medical oncology or radiation oncology colleagues? *[probe on specifics as needed]*

21) From your experience, how do your medical or radiation oncology view omission of axillary evaluation in this population? *[probe on how this is similar or different from surgical view]*

22) Who is usually part of the decision-making process around axillary evaluation?

-For example, do you think your medical or radiation oncology colleagues would like to weigh in on this decision?

23) How, if at all, would decisions around adjuvant treatment change with the omission of axillary evaluation?

© 2022 Minami CA et al. *JAMA Network Open*.

eAppendix 2. Medical and Radiation Oncologist Interview Guide

Introduction

Thank you for taking the time to speak with me today. We are speaking with physicians to better understand their perspectives on axillary evaluations in older women with breast cancer. Our discussion today will help inform a survey that will explore issues and attitudes towards the possible omission of axillary evaluation in older women with early-stage hormone-positive breast cancer. There are no right or wrong answers in our discussion today, as we are here to better understand your experience and perspectives on the topic at hand.

This interview will be recorded and takes, on average, twenty minutes. Recordings will not be shared with your colleagues, patients or families, or with anyone else outside the research team. I want to be sure that you understand that being part of this interview is voluntary, you can opt to not answer questions or end the conversation at any time.

We appreciate your participation in this study.

Obtain informed consent

Do you have any questions before we begin the interview?

Begin recording

I. Demographic Information

First off, could I get some baseline information about you?

1) How many years have you been in practice?

2) How would you describe your practice with regards to being academic or community-based or a hybrid model?

3) In what field/specialty, if any, are you board certified?

4) What fellowship training do you have, if any?

5) What is the scope of your practice?

a) What percentage of your practice is comprised of breast oncology patients?

b) Approximately how many women do you see a month who are over 70, with clinically node-negative, hormone-positive breast cancer?

c) What percentage of these patients come without pathologic nodal staging?

d) Are there any other tests that you usually order if a patient who is over 70, with clinically node-negative, hormone-positive breast cancer comes in without pathological nodal staging?

For the next set of questions, I am asking you to focus specifically on the management of patients 70 and older, with clinically node-negative, hormone-receptor positive breast cancer.

II. Current Practice

A. Individual Health Professional Factors: Professional Behavior/ Professional Interactions: Communication and Influence

6) From your experience, how do your surgical colleagues approach axillary evaluation in this population? [probe on how this is similar or different from surgical view]

© 2022 Minami CA et al. *JAMA Network Open*.

7) How would you describe the communication you have, if any, regarding axillary management in this population of patients with your surgical colleagues?

-For example, is your opinion ever elicited pre-operatively by the surgeons that you work with?

8) What happens in a situation where there is disagreement about whether axillary evaluation is needed?

-have you ever found it necessary to ask the surgeon to take the patient back to the OR for axillary evaluation?

9) How would you describe the usually approach to the decisions around evaluating the axillary in this patient population?

-Who is usually part of the decision-making process around axillary evaluation?

-What patient and disease factors play a role in the decision to evaluate the axillary?

10) What do your colleagues think of this practice?

11) How do you feel about omitting axillary evaluation in this population?

-Are there any ways that this impacts your ability to make treatment decisions?

[probe if not discussed] How, if at all, does omitting axillary evaluation in this population affect your ability to make adjuvant treatment decisions?

-What impact do you think this recommendation will have on patient outcomes? [probe on reasons behind the impact]

B. Patient Factors

13) From your perspective, what do patients think about axillary evaluations?

-How do you think they will respond to the omission of this procedure?

-Is it an overall positive or negative for the patient?

C. Social, Political, and Legal Factors

14) How is your attitude regarding axillary evaluation in this patient population affected by external factors?

Possible probes:

-Do financial incentives or disincentives play a role?

-Or institutional pressures or regulations?

-What about malpractice environment?

© 2022 Minami CA et al. *JAMA Network Open*.

III. In Theory

A. Individual Health Professional Factors: Knowledge

15) Can you tell me about your awareness/understanding of guidelines for omitting SLNB in this patient population?

16) Have you read any data/studies that explore omission of axillary evaluation in this population? If so, what do you think of these data?

17) What do you think of the data backing their choice of omitting SLNB?

If oncologist is not aware of the trial data or SSO guideline:

We are curious about use of axillary evaluation in this patient population because there are data from randomized controlled trials demonstrating that axillary evaluation could be safely omitted in this population without compromising survival, and with an increase in axillary recurrence rate of only 3%. In 2016, the Society of Surgical Oncology, the SSO, released a guideline counseling against routine use of axillary evaluation in this patient population.

18) What are your initial reactions to hearing this information?

How, if at all, does this information impact your consideration around axillary evaluation?

19) Can you describe any factors or reasons that might keep you from supporting omission of axillary evaluation in this population?

If oncologist IS aware of trial data or SSO guideline:

20) To what extent do you agree or disagree with the Society of Surgical Oncology's Choosing Wisely 2016 recommendation: "Don't routinely use sentinel node biopsy in clinically node negative women $\geq$70 years of age with hormone positive invasive breast cancer?"

Probes:

a) What were your initial reactions to this recommendation?

-What do you think of the quality of evidence supporting this practice?

b) What do you think of the clarity of the recommendation?

-How would you interpret this recommendation?

21) What is your opinion of the SSO as a source for practice recommendations?

© 2022 Minami CA et al. *JAMA Network Open*.
